# Application of Digital Analysis for Assessment of Coronary Sub-Occlusions in Autopsy Pathology: It Is Time to Move beyond Histology Alone

**DOI:** 10.3390/diagnostics14192115

**Published:** 2024-09-24

**Authors:** Giuseppe D’Abbronzo, Renato Franco, Cecilia Salzillo, Carlo Pietro Campobasso, Maurizio Municinò, Alessandro Feola, Andrea Ronchi

**Affiliations:** 1 Pathology Unit, Department of Mental and Physical Health and Preventive Medicine, University of Campania Luigi Vanvitelli, 80138 Naples, Italy; giuseppe.dabbronzo@unicampania.it (G.D.); renato.franco@unicampania.it (R.F.); andrea.ronchi@unicampania.it (A.R.); 2 PhD Course in Public Health, Department of Experimental Medicine, University of Campania “Luigi Vanvitelli”, 80138 Naples, Italy; cecilia.salzillo@unicampania.it; 3 Department of Experimental Medicine, University of Campania “Luigi Vanvitelli”, 80138 Naples, Italy; carlopietro.campobasso@unicampania.it; 4 Forensic and Legal Medicine Center, San Giuliano Hospital, 80014 Giugliano in Campania, Italy; municinodrmaurizio@gmail.com

**Keywords:** sudden cardiac death, coronary artery disease, autopsy

## Abstract

**Background:** Coronary artery disease (CAD) underlies most cases of myocardial infarction (MI), causing or at least contributing to oxygen supply–demand mismatch and myocardial injury, so a careful and reliable evaluation of the main coronary arteries and large branches is a key moment of autopsy in order to establish the cause of death. The aim of this study is to evaluate the application of digital image analysis in the assessment of coronary artery sub-occlusions. **Methods:** A total of 50 coronary sections sampled during 11 consecutive autopsies, regardless of the cause of death, were analyzed. The ideal lumen and the percentage of the residual lumen were evaluated by digital pathology using QuPath v 4.3 and by an expert pathologist. The evaluations performed were compared using Lin’s concordance correlation coefficient. **Results:** The Lin agreement index between the two evaluation methods for all measurements showed an excellent agreement rate [0.923, with confidence interval (0.866, 0.956)]. However, in the case of critical stenosis, from 60% to 80% and from 65% to 75%, the Lin agreement index between the two evaluation methods was, respectively, 0.798 [0.603, 0.904], corresponding to good agreement, and 0.516 [0.071, 0.725], corresponding to slight agreement. The digital system has superior performance in cases where lumen occlusion falls between 60% and 80% and provides an objective assessment of the residual lumen area. **Conclusions:** According to the widespread availability and ease of use of these technologies, we suggest that image analysis should be considered a routine tool and established as the diagnostic gold standard in this field.

## 1. Background

Establishing the cause of death is one of the primary goals of autopsy with obvious implications on subsequent diagnostic and medico-legal reasoning. Coronary artery disease (CAD) underlies most cases of myocardial infarction (MI), causing or at least contributing to oxygen supply–demand mismatch and myocardial injury [1], so a careful and reliable evaluation of the main coronary arteries and large branches is a key moment of autopsy, for two main reasons [2]. Firstly, it is well known that early-onset acute myocardial infarction is not detectable by conventional histology of the myocardium. If death occurs within a few hours of the acute MI, the histological examination fails to identify any specific diagnostic features and consequently, the diagnosis relies mainly on the evidence of the coronary artery occlusion. Secondly, the evaluation of coronaries plays a fundamental role in the epicrisis of sudden unexplained death classified as deaths of arrhythmic origin, since hemodynamically significant occlusion of one or more coronary arteries is a known risk factor for the onset of an acute arrhythmia, mainly ventricular tachyarrhythmia triggered by acute myocardial ischemia [3,4]. Each autopsy requires an extensive inspection of the coronary arteries, through gross sectioning and gross evaluation of the vessels to detect, locate, and quantify any occlusion or sub-occlusion, due to atherosclerotic plaques or thrombi. Multiple transverse cuts at 3 mm intervals along the course of the main epicardial coronary arteries are recommended, and the most severe focal lesions should be sampled for histology [5]. In this context, one of the main purposes of the histological examination in this setting is the precise quantification of the coronary occlusion. The correct quantification of the coronary occlusion is mandatory, since the occlusion is considered hemodynamically significant if it affects more than 70% of the lumen, and a high-risk plaque is defined by a residual patent luminal area smaller than 4 mm^2^ [6]. According to the guidelines for autopsy investigation of sudden cardiac death (SCD), a coronary lumen stenosis >75% is considered enough evidence that can support the diagnosis of SCD as highly probable [5]. If we consider that coronary alterations may be the only detectable pathological feature in sudden unexpected deaths, the evaluation takes on even greater importance as it may represent the only objective source of information in such cases. However, the assessment and quantification of coronary occlusion through both gross and histological evaluation is reliant upon the operator, being subject to interindividual interpretative variability. Moreover, the assessment of the exact lumen occlusion percentage may be challenging for the pathologist and measuring the patent lumen surface can be almost impossible, such that it is not performed in daily practice. In the last few years, the development of digital pathology has been revolutionizing the world of pathology. Indeed, the possibility to digitize histological images has allowed the development of digital image analysis software, offering an objective and reproducible assessment of specific morphological findings [7]. Although digital pathology is widely applied in many fields of surgical pathology [8], its application to autopsy pathology has not yet been evaluated.

The aim of this study was to evaluate the application of digital image analysis in the assessment of coronary artery sub-occlusions. We propose to include digital image analysis in the standard protocol for the histological evaluation of coronary occlusion on post-mortem samples.

## 2. Materials and Methods

### 2.1. Cases Selection

This study included all the coronary sections sampled during 11 consecutive autopsies performed between 1 September 2023 and 1 January 2024, regardless of the cause of death. Fifty sections were included in the study.

### 2.2. Coronary Artery Gross Evaluation and Sampling, Histology, and Ancillary Technique

**Figure 1:**
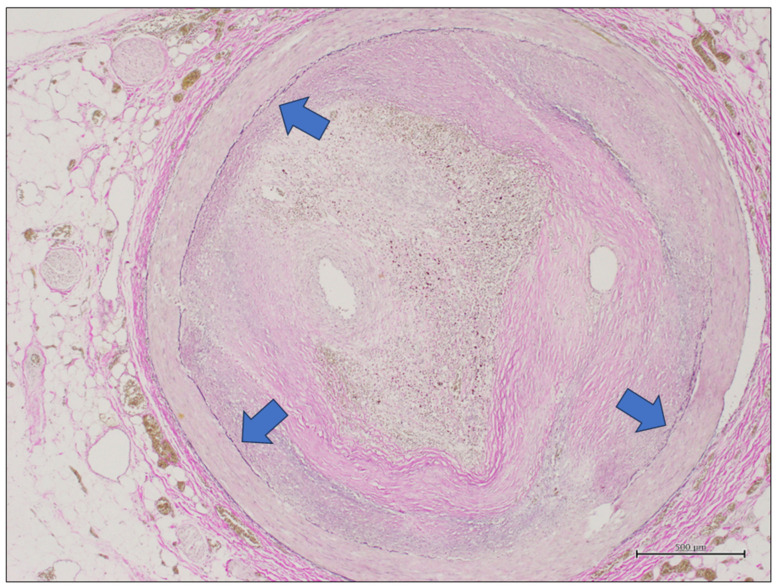
A coronary artery section stained by elastic stain. The internal elastic membrane is highlighted by this stain as it is made up of elastic fibers and appears as a black circumference (blue arrows). This circumference defines the ideal lumen of the vessel, defined as the circumference corresponding to the vessel lumen in the absence of plaque (elastic stain, original magnification 20×).

In all cases, the gross evaluation and sampling of coronary arteries were carried out according to the recommended autopsy technique [5]. Particularly, the main coronary arteries were sectioned with transversal parallel cuts at 3 mm intervals, and the vessel segments with more evident gross signs of occlusion were sampled for histological examination. The samples were fixed in formalin and included in paraffin, as routinely performed in pathology laboratories. Heavily calcified arteries were decalcified prior to cross-sectioning. Five µm thickened tissue sections were cut from each paraffin block and stained by hematoxylin and eosin. A further 5 µm thickened tissue section from each paraffin block was stained by elastic histochemical stain. This staining was chosen because it allows us to have information on the ideal lumen of the vessel, highlighting the internal elastic membrane of the vessel (Figure 1). The elastic stain was performed automatically using an Elastic Stain Core Kit (Ventana-Roche) on a BenchMark ULTRA instrument (Ventana-Roche).

### 2.3. Digital Pathology

**Figure 2:**
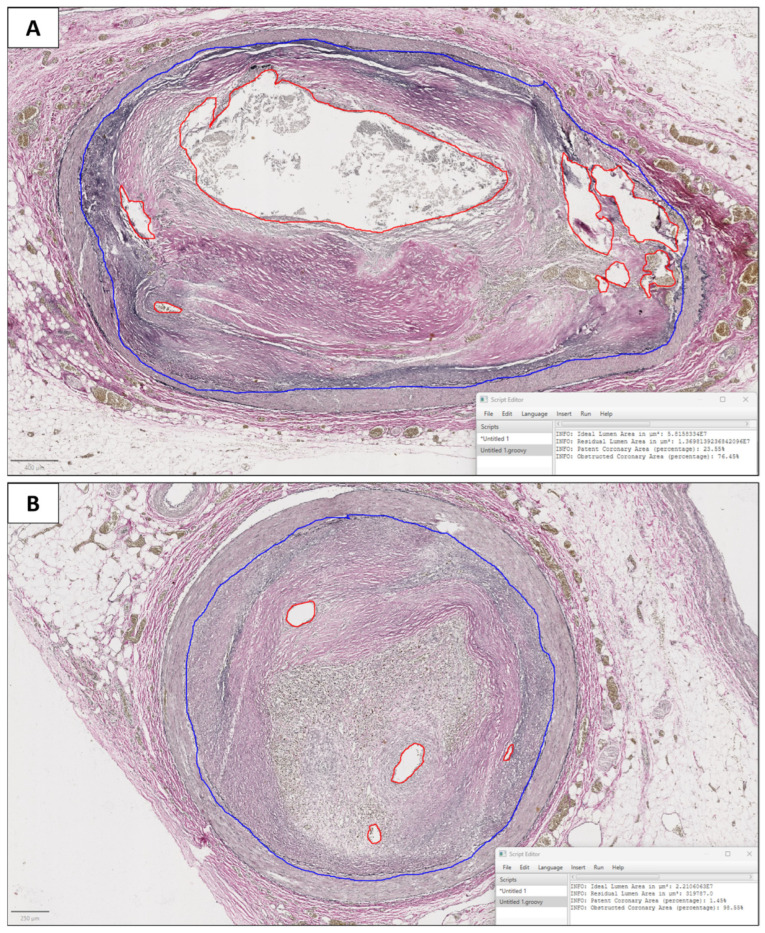
Two coronary artery sections (A: subject J, section 43; B: subject K, section 47) analyzed by digital image analysis. The blue line corresponds to the ideal lumen (internal elastic membrane), and the red line corresponds to the residual lumen. In (**A**), the effective lumen of the vessel included a part of the lumen spared by the plaque, and some recanalization channels within the plaque. In (**B**), the residual lumen only included some recanalization channels within the plaque. In the right bottom, the software automatically calculated the area of both the ideal and the residual lumen, and the percentage of the lumen stenosis (A: 76.45%; B: 98.55%).

A histological slide stained by elastic stain was scanned for each case with a Ventana DP200 Slide Scanner and exported as whole-slide images (WSIs) in tiff. Subsequently, WSIs were uploaded in QuPath v 4.3 [9] and a trained pathologist conducted annotation, drawing the ideal lumen area and residual lumen area (Figure 2). We defined the ideal lumen as the circumference corresponding to the vessel lumen in the absence of plaque (corresponding to the internal elastic membrane), and the residual lumen as the lumen space effectively left free by the plaque. Then, we developed a specific script for QuPath, in Groovy language, that calculated the residual lumen area and ideal lumen area. Then, our script calculated the percentage of the residual lumen compared to the ideal lumen as the ratio between the residual lumen area and ideal lumen area; finally, it calculated the percentage of lumen occlusion (*LO*) according to the formula:

*LO* = 100 − *percentage of residual lumen*.

### 2.4. Pathologist Evaluation

Each coronary artery section was evaluated by a pathologist with experience in autopsy and cardiac pathology. The pathologist evaluated each section stained by hematoxylin and eosin and by elastic stain through light microscopy and assessed the percentage of stenosis.

### 2.5. Statistical Analysis

We compared the evaluation performed by an experienced pathologist with the evaluation performed by the software using Lin’s concordance correlation coefficient [10]. Lin’s concordance correlation coefficient (*CCC*) considers both the correlation of the assessments (Pearson correlation) and the average and variance of the measurements:

$$CCC:\;\frac{2p\sigma_{x}\sigma_{y}}{\sigma_{x}^{2}+\sigma_{y}^{2}+{\left(\mu_{x}+\mu_{y}\right)}^{2}}$$

*p*: Pearson correlation coefficient;

$\sigma_{x}^{2};\;\sigma_{y}^{2}$: variance of measurement;

$\mu_{x};\;\mu_{y}$: average of measurement.

Thus, the agreement between the software and pathologist penalizes both systematic and random differences.

### 2.6. Hardware and Software

Statistical analyses were performed using a specific script developed for Python ver. 3.10.12 (using the stats template library) using the Anaconda^®^ distribution, with Jupyter Notebook as the programming environment. All computing procedures were performed on Alienware Aurora^®^ R11 hardware with an Intel (R) Core (TM) i5-10600KF CPU at 4.10 GHz, 64 GB of RAM, and an AMD Radeon RX 5700 XT graphics card with 8 GB of dedicated memory.

## 3. Results

### 3.1. General Features of the Series

Eleven subjects submitted to autopsy were included in the study, including eight males and three females, with their age ranging from 29 years to 83 years (mean age: 62.4 years; median age: 62 years). Most of them were natural deaths except for three cases of violent deaths. Details of each case including age, sex, cause of death, and level of coronary atherosclerosis are reported in Table 1.

Regarding fatal arrythmia as death’s causes, it is known that fatal arrhythmias can be a consequence of ischemic heart disease due to uncomplicated atherosclerosis [11]. In our study, necroscopic examination excluded other causes of sudden death (i.e., drug overdose, cardiomyopathy, and cardiac hypertrophy) so we considered death by fatal arrythmia as a consequence of ischemic heart disease due to atherosclerosis.

### 3.2. Digital Pathology Analysis

All 50 coronary artery sections were adequate for the digital analysis. The software calculated the area of both the ideal and the residual lumen, and the percentage of the lumen stenosis for each section. The percentage of the lumen stenosis evaluated by the pathologist’s eye ranged from 30% to 99% (median value: 75%; mean value: 70.8%). The percentage of the lumen stenosis evaluated by digital image analysis ranged from 35.2% to 99% (median value: 73.3%; mean value: 71.9%). The results are reported in Table 2.

Two examples of digital image analysis are showed in Figure 2.

### 3.3. Statistical Analysis

Overall, the Lin agreement index between the two evaluation methods for all measurements in the dataset was 0.923 (rounded to three decimal places), with a confidence interval [0.866, 0.956], corresponding to excellent agreement. Regarding the coronary stenosis in the critical ranges, from 60% to 80% and from 65% to 75%, the Lin agreement index between the two evaluation methods was, respectively, 0.798 [0.603, 0.904], corresponding to good agreement, and 0.516 [0.071, 0.725], corresponding to slight agreement (Table 3).

## 4. Discussion

Assessing coronary arteries is critical to understanding the pathological mechanism leading to an individual’s death. Indeed, beyond those who died violently, a significant number of autopsied individuals have been reported to have died of cardiac causes [12]. Among presumed cases of SCD, coronary disease stands as the leading cause, accounting for approximately 32% of cases, followed by fatal arrhythmia due to unknown drug overdose, cardiomyopathy, and cardiac hypertrophy [13]. Accurate assessment of the coronary arteries is crucial for two primary reasons. Firstly, conventional histological examination of the myocardium often fails to detect early-onset acute myocardial infarction. Therefore, the diagnosis heavily relies on the identification of the coronary artery occlusion. Secondly, evaluating the coronary arteries is pivotal in the post-mortem analysis of SCD categorized as arrhythmic. Indeed, hemodynamically significant occlusion in one or more coronary arteries is a recognized risk factor for triggering fatal arrhythmias, particularly ventricular tachyarrhythmia induced by acute myocardial ischemia. However, in every case of SCD, it is essential to include not only the examination of the various coronary branches but also histologic and immunohistochemical analysis of the myocardium of both ventricles, by standard sampling and targeted sampling of evident and suspicious lesions with myocardial morphology [14,15]. Guidelines for the autopsy assessment of SCD include evaluating the coronary arteries through multiple transverse cuts at 3 mm intervals along the course of the main epicardial arteries. Additionally, histological evaluation of the coronary sections with the most severe gross occlusions is essential for an accurate diagnosis and determination of the cause of death [5]. The histological assessment of coronary stenosis requires meticulous precision, as sudden arrhythmic death resulting from coronary artery disease is particularly probable when stenosis is higher than 70% [6]. However, coronary stenosis is assessed visually through a microscope examination of the vessel section, depending on the pathologist’s judgment. As such, this specific assessment requires specific training for pathologists, but it is invariably affected by interindividual variability. In recent years, digital pathology has introduced more objective and reproducible evaluation methods across various fields of pathological anatomy [16,17,18]. This difficulty is compounded by the fact that atherosclerotic plaque typically does not originate from a single point on the arterial wall and extend to occlude only a portion of the lumen’s circumference. Instead, it often affects a significant portion or the entirety of the lumen’s circumference before growing inward. Therefore, an accurate definition of the area between two concentric circles (referred to as “annulus” in geometry) is quite challenging to carry out by visual inspection. Moreover, the residual lumen of a vessel partially occluded by plaque often consists not of a single lumen but of a combination of recanalization channels within the plaque, as observed in Figure 2. For all these reasons, the histological evaluation of coronary arteries requires pathologists with specific training, which may not always be readily available at all centers. In this study, we suggest an image analysis procedure that any pathologist may apply to provide an objective, rapid, and precise assessment of coronary stenosis on histological sections. The histochemical staining for elastic fibers enabled the clear identification of the internal elastic membrane, corresponding to the ideal vessel lumen. Subsequently, the stained histological sections were scanned and analyzed using the image analysis system, providing an accurate measurement of the percentage of vessel stenosis. Statistical analysis demonstrated excellent agreement with an expert pathologist in the field. However, the agreement between the digital system and the expert pathologist was lower for luminal stenosis values between 60% and 80% (0.798, indicating substantial agreement), and even lower for values between 65% and 75% (0.516, indicating poor agreement). The exact definition of coronary stenosis is particularly important in such cases, as a lumen stenosis of 70% defines the greatest risk of acute events [6]. Moreover, the evaluation performed by the digital system provided precise measurements to the decimal point and also included an assessment of the residual lumen area. This additional information is crucial, as a residual lumen area smaller than 4 mm^2^ has been associated with a higher risk of sudden death [6]. It is noteworthy that within the critical threshold for coronary occlusion (>70%), discrepancies exist between the digital evaluations and those performed by the pathologist in eight sections (15, 22, 28, 30, 39, 40, 41, 49). In five of these cases (15, 22, 30, 41, 49), the digital analysis provides a greater assessment of the extent of occlusion compared to the pathologist’s assessment, classifying the sections as being within the critical range. Conversely, in three cases (28, 39, 40), the digital assessment provides a lesser evaluation of the severity, placing these sections in a lower-risk category. Additionally, in a singular instance, the analysis of a specific coronary section (section 22, subject F, deceased due to arrhythmia) altered the risk classification from a low-risk category (65% occlusion) to a high-risk category (70% occlusion). This assessment may provide further insights into the probable etiology of the fatal arrhythmia, which is likely secondary to chronic ischemia. In the study by Ford et al. (2001), twenty randomly selected Movat-stained cross-sections of coronary arteries were independently reviewed three times, with a three-month interval, by six clinical pathologists, six pathology residents, seven anatomic pathologists, and two cardiovascular pathologists [19]. Before the third review, all participants underwent training in coronary artery stenosis assessment. The study revealed suboptimal diagnostic performance in the histological evaluation of coronary stenosis, with sensitivity ranging from 0.48 (for anatomic pathologists) to 0.61 (for clinical pathologists) after training. In addition, the analysis indicated that stenotic arterial cross-sections with residual lumens displaying concentric or eccentric polymorphous shapes were consistently underestimated compared to a proposed image analysis. Conversely, lumens with an eccentric slit-like shape were consistently overestimated [19]. Interestingly, the authors of this study utilized image analysis to precisely assess the percentage of luminal stenosis. However, for their analysis, Ford et al. (2001) could only access photographic images [19]. Therefore, they adopted an indirect method to calculate the areas of the ideal and actual lumens of the coronary arteries. This method was based on the measurement of the analyzed sections’ radius through an automated system, then calculating the circumference using the formula C = 2πR and the area with A = πR^2^. Based on this approach, they assumed that the analyzed surfaces corresponded to perfect circles. In our study, we adopted a different approach by utilizing advanced software, QuPath, which allowed us to directly calculate the area of the regions of interest (the ideal lumen and the residual coronary lumen) by analyzing whole-slide images (WSIs). Recently, Barth et al. developed two computer-generated morphometric guides to estimate the degree of luminal narrowing in atherosclerotic coronary arteries [20]. One guide is based on symmetric or eccentric circular or crescentic narrowing, while the other focuses on slit-like or irregularly shaped narrowing. Barth et al. (2017) created digital whole-slide images of 20 cross-sections of the left anterior descending (LAD) coronary artery and developed visual guides using Adobe Photoshop CS5. Four pathologists independently reviewed and scored the degree of luminal narrowing using these visual guides. Interobserver reliability was high, with intraclass correlation coefficients of 0.874 and 0.899 for H&E- and Movat-stained sections, respectively. The visual scores were, on average, approximately 8% lower than the morphometric assessment (52.7% vs. 60.2%) [20]. In conclusion, in cases of suspected SCD, whether the autopsy findings deal with “coronaric” or “myocardial” lesions and arrhythmic or non-arrhythmic mechanisms, there may be a variety of possible combinations that need to still be investigated before the final diagnosis of SCD can be formulated [21]. Based on the results of this research study, the digital system outperforms expert pathology for several reasons. Firstly, it allows for a more precise evaluation. Secondly, it demonstrates superior performance in cases where lumen occlusion falls between 60% and 80%. Lastly, it provides an objective assessment of the residual lumen area. Despite the satisfactory results, our study presents some limitations that highlight the need for further research: firstly, the small sample size calls for an expansion to strengthen the robustness of the results. Additionally, the patients included in the study were affected by different pathologies; therefore, it would be advisable to harmonize the sample. Once the sample size is increased, it would be beneficial to stratify the cases into more homogeneous groups to ensure greater consistency and reliability of the findings.

Furthermore, as has already been proposed for other technologies used in post-mortem examinations [22,23], it would be important to establish a precise protocol for the standardized use of the digital system evaluation of coronary obstruction, defining parameters such as fixation times, sample preparation, and the application of histological staining protocols. Moreover, our digital system could be implemented with an AI-based system capable of recognizing the area of the ideal lumen and the area of the residual lumen for the automated drawing of the areas of interest.

Overall, these data emphasize the importance of utilizing a digital image analysis system for the accurate and efficient evaluation of coronary stenosis in post-mortem histological samples.

Considering the widespread availability and ease of use of these technologies, we argue that image analysis should be considered a routine tool and established as the diagnostic gold standard in this field.

## Figures and Tables

**Table 1:** Details of the autoptic cases included in the series. Abbreviations: m: male; f: female; DIC: disseminated intravascular coagulation; SCD: sudden cardiac death.

Autoptic Cases Included in the Series				
Subject	Sex	Age	Cause of Death	Coronary Atherosclerosis
A	m	51	Burns, sepsis, DIC	Fibrosis plaque (uncomplicated atherosclerosis)
B	f	83	Hypothermia	Initial atherosclerosis
C	f	64	Subarachnoid haemorrhage	Fibrosis plaque (uncomplicated atherosclerosis)
D	m	62	SCD due to arrhythmia	Fibrosis plaque (uncomplicated atherosclerosis)
E	m	59	SCD due to arrhythmia	Complicated atherosclerosis
F	f	76	SCD due to arrhythmia	Fibrosis plaque (uncomplicated atherosclerosis)
G	m	77	Polytrauma, DIC	Complicated atherosclerosis
H	m	29	Myocardial infarction	Complicated atherosclerosis
I	m	76	Polytrauma, DIC	Fibrosis plaque (uncomplicated atherosclerosis)
J	m	53	SCD due to arrhythmia	Fibrosis plaque (uncomplicated atherosclerosis)
K	m	56	Myocardial infarction	Complicated atherosclerosis

**Table 2:** Details of the results.

Percentage of Lumen Stenosis Evaluated by Eye Versus Digital Image Analysis			
Subject	Section	Eye (%)	Digital (%)
A	1	55	65.6
	2	50	60
	3	55	65
	4	85	83
B	5	35	41.6
	6	35	35.2
C	7	50	54
	8	30	37.6
	9	40	42.1
D	10	75	77.8
	11	80	84.1
	12	65	57.8
	13	85	82.2
	14	80	83.6
	15	70	76.7
	16	50	54
E	17	90	91
	18	85	91.6
	19	80	79.6
	20	95	91.6
	21	40	53.4
F	22	65	71.4
G	23	45	53
	24	47	55.5
H	25	92	85.7
	26	80	81.8
	27	82	76.1
	28	83	66.4
	29	77	75.9
	30	55	73.3
	31	75	71.7
I	32	65	64.2
	33	92	82
	34	62	62.6
	35	62	62.7
	36	90	87.1
	37	82	71
	38	88	82.8
	39	78	68.3
J	40	70	66
	41	70	73.1
	42	80	78.6
	43	80	76.5
	44	75	71.8
K	45	99	99
	46	98	96.9
	47	98	98.6
	48	90	94.5
	49	65	73.5
	50	65	67.7

**Table 3:** Lin agreement index between eye evaluation and digital evaluation.

Lin Agreement Index between Eye Evaluation and Digital Evaluation	
Lumen Stenosis Range	Lin Agreement Index
Overall	0.923
60–80%	0.798
65–75%	0.516

## Data Availability

Additional data can be requested from the corresponding author.
